# Methodological issues associated with collecting sensitive information over the telephone - experience from an Australian non-suicidal self-injury (NSSI) prevalence study

**DOI:** 10.1186/1471-2288-11-20

**Published:** 2011-02-17

**Authors:** Anne W Taylor, Graham Martin, Eleonora Dal Grande, Sarah Swannell, Simon Fullerton, Philip Hazell, James E Harrison

**Affiliations:** 1 Population Research and Outcome Studies, School of Medicine, University of Adelaide, Adelaide (5000), Australia; 2 Child and Adolescent Psychiatry, The University of Queensland, Queensland (4072), Australia; 3 Centre for Suicide Prevention Studies, The University of Queensland, Queensland (4072), Australia; 4 Sydney Medical School, The University of Sydney, Sydney (2006), Australia; 5 Research Centre for Injury Studies, Flinders University, Adelaide (5042), Australia

## Abstract

**Background:**

Collecting population data on sensitive issues such as non-suicidal self-injury (NSSI) is problematic. Case note audits or hospital/clinic based presentations only record severe cases and do not distinguish between suicidal and non-suicidal intent. Community surveys have largely been limited to school and university students, resulting in little much needed population-based data on NSSI. Collecting these data via a large scale population survey presents challenges to survey methodologists. This paper addresses the methodological issues associated with collecting this type of data via CATI.

**Methods:**

An Australia-wide population survey was funded by the Australian Government to determine prevalence estimates of NSSI and associations, predictors, relationships to suicide attempts and suicide ideation, and outcomes. Computer assisted telephone interviewing (CATI) on a random sample of the Australian population aged 10+ years of age from randomly selected households, was undertaken.

**Results:**

Overall, from 31,216 eligible households, 12,006 interviews were undertaken (response rate 38.5%). The 4-week prevalence of NSSI was 1.1% (95% ci 0.9-1.3%) and lifetime prevalence was 8.1% (95% ci 7.6-8.6).

Methodological concerns and challenges in regard to collection of these data included extensive interviewer training and post interview counselling. Ethical considerations, especially with children as young as 10 years of age being asked sensitive questions, were addressed prior to data collection. The solution required a large amount of information to be sent to each selected household prior to the telephone interview which contributed to a lower than expected response rate. Non-coverage error caused by the population of interest being highly mobile, homeless or institutionalised was also a suspected issue in this low prevalence condition. In many circumstances the numbers missing from the sampling frame are small enough to not cause worry, especially when compared with the population as a whole, but within the population of interest to us, we believe that the most likely direction of bias is towards an underestimation of our prevalence estimates.

**Conclusion:**

Collecting valid and reliable data is a paramount concern of health researchers and survey research methodologists. The challenge is to design cost-effective studies especially those associated with low-prevalence issues, and to balance time and convenience against validity, reliability, sampling, coverage, non-response and measurement error issues.

## Background

Torangeau and Smith [1] determined that a sensitive survey question is one that 'raises concerns about disapproval or other consequences (such as legal sanctions) for reporting truthfully or if the question itself is seen as an invasion of privacy'. Collecting representative data on sensitive issues, especially data that are used to determine population prevalence estimates, is problematic whatever mode of data collection is employed [2-6]. Surveys (personal computer, telephone, web or paper based) are commonly used to collect these data but respondents can feel threatened or stressed when answering questions about highly personal, private, often illegal or embarrassing, socially stigmatising, and sometimes sacred issues. Many sensitive behaviours are confidential, and not shared, even among those close to the person. Although questions on sexual behaviours and religion are now more acceptable in surveys, history of child abuse, drug habits, intimate partner violence and abortion experiences are still considered to be sensitive [7,8].

One of the sensitive issues surfacing as a priority in recent years is non-suicidal self-injury (NSSI), resulting in this important public health issue receiving increased prominence [9]. NSSI includes deliberate destruction or alteration of body tissue without suicidal intent and includes (but is not limited to) cutting, scratching, burning, and punching, hitting or slapping [10]. It is prevalent during adolescence [11,12]. NSSI causes distress for individuals, their family and friends and can be costly for the health system when it escalates into serious harm. Many individuals are treated badly by professionals due to the stigmatisation of self-injury as a purely attention seeking and manipulative behaviour [13,14]. NSSI is not a suicide attempt *per se*, but of most concern is the strong relationship between self-injury and suicide attempts with previous occasions of NSSI a strong risk factor for suicide [9,12,15].

As with other sensitive issues, collecting prevalence data on NSSI is challenging. As well as problems associated with case definition [12,15,16] official figures based on hospital presentations or hospital clinics routinely underestimate the magnitude of the problem with only a small proportion of people with NSSI reaching a hospital or clinical setting [13,15,16]. Much of the other previous research has been limited to school-aged children with data collected using self-report questionnaires often administered by school teachers or school nurses [12,14,16-20]. Hargus argues that self-report questionnaires completed by adolescents are vulnerable to their 'whims, interpretations, perspectives, and self-awareness' [12]. School-based studies also have the possibility of inaccurate reporting based on the close proximity of peers, issues with selected samples (e.g. schools) and method of delivery (school nurses). A large number of university base studies have also been undertaken [21-23].

An Australian-wide survey was funded by the Australian Government's Department of Health and Ageing to determine the prevalence of NSSI in the Australian community, and the methods people use to self-injure. The Australian National Epidemiological Study of Self-injury (ANESSI) was designed to overcome problems associated with previous research such as the varying modes of collection, sample age ranges and time frames, and the limited number of putative risk factors for NSSI that have been examined. ANESSI was designed to explore relationships between self-injury and suicide attempts, suicidal ideation, mental health, substance use, trauma and the nature of deliberate self-injury. In addition, ANESSI explored predictors, underlying motives and outcomes of NSSI including associated treatment patterns. Computer assisted telephone interviewing (CATI), on a sample of the Australian population aged 10+ years, was the method of choice based on cost, coverage, ease of administration, flexibility of the system and time frame of the project.

This paper only addresses the methodological issues highlighted in collecting this sensitive information. Other publications have provided results and major associations and relationships [24,25].

## Methods

Prior to the main survey, a pilot study of 50 randomly selected households was conducted to test question formats and sequence, and to assess survey procedures. To ensure adequate pilot testing of each component of the questionnaire, it was also piloted with volunteers who currently, or had previously, self-injured. These volunteers were current or ex-patients of one of the researchers, or had previously taken part in a qualitative study about self-injury. The questionnaire was amended at both pilot stages based on the information obtained. The final questionnaire included the GHQ12 [26], questions on emotion regulation, alexithymia, coping, family functioning, dissociation, psychiatric history, trauma, sexual orientation, service use, substance use and a range of demographics. The suicide ideation questions asked were four questions from the GHQ28 [27] that have previously shown validity [28]. The NSSI questions included current and past activity, method of NSSI, level of pain, reason why, age of first NSSI, help seeking behaviour, level and frequency of medical treatment, and intention and success of reducing or stopping NSSI.

All households in Australia with a telephone connected and the telephone number listed in the Australian Electronic White Pages (EWP) were eligible for random selection. An approach letter was sent to all selected households detailing the purpose of the study and notifying the household they would receive a telephone call. Along with the letter there was an information sheet regarding the study and self-injury, a list of mental health services, a list of Indigenous health contacts and a summary of the questions that would be asked in the survey.

Within each contacted household the person, aged 10 years or over, and last to have a birthday, was randomly selected. There was no replacement for non-contactable persons. The birthday method of selection provides all household members an equal chance of being selected, no working numbers are wasted, it is easy to administer and does not result in a higher number of older people being selected. Another advantage of this method, is that it does not involve asking sensitive questions early in the interview [29]. It has also been suggested that interviewers prefer the last birthday method because "it does not make the respondent uneasy" [29] and it does not cause problems for women living on their own.

A minimum of 10 call-backs were made to telephone numbers selected to interview household members. Different times of the day or evening were scheduled for each call-back. If the person could not be interviewed immediately they were re-scheduled for interview at a time suitable to them. Replacement interviews for persons who could not be contacted or interviewed were not permitted. If the selected person was a child under 18 years of age, parental (or caregiver) permission was first gained. If the child felt uncomfortable about being interviewed, permission for the adult/caregiver to answer the questionnaire on their behalf was sought.

Data collection was undertaken by the contracted agency from January to July 2008. On initial contact with each household, the interviewer identified themselves and the purpose of the survey. If required, appointments were made to conduct the interview in Italian, Greek, Vietnamese, Chinese, or Arabic. Interviews were conducted using CATI methodology. This methodology allows immediate entry of data from the interviewer's questionnaire screen to the computer database. The main advantages of this system are the precise ordering and timing of call backs and correct sequencing of questions as specific answers are given. The CATI system enforces a range of checks on each response with most questions having a set of pre-determined response categories. In addition, CATI automatically rotates response categories, when required, to minimise bias. When open-ended responses were required, these were transcribed verbatim by the interviewer.

Extensive interviewing training was undertaken including interactive information sessions conducted by the study team detailing the background to the study and the sort of issues that may arise. In addition, only interviewers who were comfortable with the survey topic, and the possibility of hearing some distressing and unpleasant details, were selected for undertaking the interviews. If for any reason an interviewer declined to accept working on this project, there were no repercussions or negative connotations for their employment situation. All interviewers were offered counselling at any stage during the six months of data collection and although confidentiality was paramount, interviewers were encouraged to debrief with their supervisor at the end of each session of interviewing. For validation purposes, the supervisor randomly checked the total number of interviews conducted by an interviewer on an ongoing daily basis and listened in, using special headphones, to 10% of each interviewers work. Data from the CATI system were imported into SPSS version 17.0. The data were weighted by age, sex and state to reflect the structure of the Australian population ten years and over. This was used to correct for areas of disproportion within the sample with respect to the population of interest and unequal sample inclusion probabilities.

The project was carried out according to the National Statement on Ethical Conduct in Research Involving Humans produced by the National Health and Medical Research Council (NHMRC) of Australia and was approved by the Behavioural & Social Sciences Ethical Review Committee of The University of Queensland. Ethical considerations were treated very seriously in this project. The ethics committee required that extensive information was sent to potential respondents before the interview. This comprised an approach letter, a three page summary overview of survey questions, two-page participant information sheets for adults and for children, an extensive list of Indigenous health contacts in each state and territory of Australia, and a list of mental health contacts (five pages in total).

As some of the questions included in the survey had the potential to cause distress or concern during the interview, telephone numbers of relevant support services were offered to respondents in the approach letter as well as at the end of the telephone interview. Use of a method involving spoken communication helped to ensure that this information was brought to the attention of all interviewees, and allowed for the option of ceasing an interview sensitively if a subject became distressed.

The ethical issues associated with seeking information about deliberate self-injury and suicidal acts by people younger than 18 years required special care. Since NSSI often arises at ages under 18 years it was essential to the purposes of the project to obtain information concerning people in this age group. The following protocol was designed to achieve this and be ethically acceptable. When the sampled person was aged under 18 years, his or her parent (or other responsible adult caregiver) was asked to give permission for the child to be interviewed. If the adult gave permission, then the child was asked whether he or she was willing to be interviewed. If the adult consented to the interview, but the child did not want to be interviewed, then the child was asked to give permission for the adult to answer the questions on the child's behalf. If the adult did not give permission for the child to answer the questions, then the adult was asked if he or she was willing to answer the questions on behalf of the child. This time-consuming and therefore costly protocol was worthwhile.

## Results

In total, 12,006 interviews were conducted. The overall sample response rate was 38.5%. On average, interviews took 13.6 minutes to complete. Of the 31,216 eligible telephone numbers, 48.5% of households refused to take part. However, once engaged, only n = 173 terminated the interview part way through. The mean age of respondents was 42.19 (range 10 to 100). Overall, 583 (4.9%) of interviews were with children under the age of 18 years and 50.5% of respondents were female. The 4-week prevalence of self-injury was 1.1% (95% ci 0.9-1.3) and lifetime prevalence was 8.1% (95% ci 7.6-8.6). Among those who had ever self-injured, 32.9% had previously attempted suicide, compared to 2% of those who had never self-injured. Overall 83% of those who had self-injured in the previous month did not receive medical attention.

## Discussion

This study provided, for the first time in Australia, population-wide prevalence estimates for NSSI. The data are valuable because they improve understanding of the extent, nature and associations of NSSI in Australia, which strengthens the case for increased acceptance of self-injury as a priority health issue, for which preventive strategies are required. While the results are valuable, the data collection stage was an expensive and time consuming activity with many methodological issues and concerns compounded by the sensitive nature of the topic. Detailed below are the main lessons learnt and the challenges faced.

### Lessons learnt

Data required for this study could have been collected using a range of survey modes including self completion via written questionnaires (delivered by post or internet), face-to-face interviews, and various computer assisted personal interviewing methods. Each has strengths and weaknesses. Although overcoming the problem of socially desirable responses crosses all methods of data collection, especially when dealing with sensitive topics, computer-assisted interviewing has long been seen as an appropriate method in which to collect sensitive survey information because of the privacy it affords [3,30]. The perceived extra level of confidentiality has ensured less non-response but it has also been argued that the extra level of privacy allows less than truthful responses to be given.

The response rate of less than 40% was acceptable for this type of survey but the potential for survey non-response bias is acknowledged. Response rates are declining in surveys based on all forms of interviewing [31,32], as people have become more active in protecting their privacy. Unacceptably low response rates, one of the main causes of non-response error, is increasingly challenging the continued success of population surveys as a means of collecting valid, representative, reliable and useful data on health (and many other issues) [33]. The growth of telemarketing has disillusioned the community and diminished the success of legitimate social science research by means of telephone-based surveys [34]. Response rates over 70% signal 'the most rigorously conducted surveys' [35] but refusals and non-contacts are the main reasons for the lowering of response rates and continue to plague the attainment of a successful interview [36,37]. In addition, the Electronic White Pages (EWP) used for this project was outdated, because of Australian privacy laws. While this may have resulted in numbers being non-contactable (telephone numbers being disconnected since publication of the EWP), the Australian telephone number provider re-allocates numbers so that these numbers may still be available (although the mail-out information would have been sent to the original address).

Studies based on sampling households from EWP are also being complicated by technical and behavioural changes. Although land-line telephones were connected to about 95% of Australian households, this proportion is decreasing with the rise of 'mobile-only' households and of internet telephone connections (i.e. VOIP). Moreover, among households with landline connections, 'silent numbers' (not listed in the EWP) and screening of calls by means of answering machines, have risen. The use of an EWP to sample from the Australian population, and the likely extent of sampling bias, have been described previously [38,39]. Weighting, as a way of correcting for non-response bias, is credited to the work of Horvitz and Thompson in the 1950s [40]. The weighting of the data (post survey weighting adjustment) corrects for non-response bias (and under-coverage) by using the weights to adjust for differences in non-probability of selection. The weights reflect unequal sample inclusion probability and compensate for differential non-response within the sample obtained. The results are representative of the variables used in calculating weights (in this study: age group by sex and state or territory of residence).

The quality of the interviewers was fundamental to the success of the telephone interviews and the quality of the answers given. Well over 99% of interviews commenced were taken to completion, despite the inclusion of items on several sensitive topics. We believe that the prior experience with CATI of the interviewers, supplemented by extensive project-specific training, gave them justified confidence in undertaking interviews on this sensitive topic. That enabled the interviewers to develop a rapport with subjects, including those who had self injured, which helped the interviewees to disclose openly, honestly and in detail to interviewers with whom they felt comfortable [41,42].

We did not put any emphasis on matching the gender of interviewer and interviewee, as recommended by Catania et al [42]. All interviews in this study were conducted by women. Special attention was paid to the vocabulary of the questionnaire and the use of non-judgemental words [3] and, where possible, yes/no responses were invited, rather than disclosure of details that perhaps could have been heard if the respondent was within earshot of another person. In addition, call-backs were scheduled to be undertaken by a different interviewer so as to achieve an increased co-operation rate [43].

### Challenges encountered

Provision of a large amount of information and in such detail as required by the ethics committee, has been shown to reduce response rate, especially for sensitive issues [31]. We have not found empirical evidence specific to the question of whether such deterrence from participation is likely to have been different for potential subjects with and without a personal history of NSSI. Those with such a history might be deterred from participating by the prospect of being asked to divulge details about their private, sometimes stressful and socially undesirable behaviour. Conversely, others might welcome an opportunity to contribute to the understanding of a problem about which they have intimate knowledge. Those without a personal history of NSSI, or close contact with it, might be deterred from participating by a perception that the survey is irrelevant to them or that they would not be able to provide useful information. In addition, detailed forewarning of the content of the survey, might increase the number of people with a history of NSSI who participate but under-report their experiences [44]. We think that the most likely net effect of these unmeasured influences is in the direction of under-estimation of the prevalence of NSSI.

Most population-based sample surveys are subject to non-coverage error. Commonly, people in hospitals, prisons, nursing homes and hotels/motels are out-of-scope for a survey method based on sampling households [33]. In many circumstances, non-coverage of a small proportion of the target population has modest impact on findings. However, the impact can be large if the proportion not covered is large, and if those who are out-of-scope differ from those who are in-scope in terms of matters important for the study. It is plausible to suppose that NSSI might be associated with being highly mobile, homeless or institutionalised, in which case non-inclusion will have resulted in underestimation of prevalence of NSSI. In addition, the inappropriateness of telephone surveys for Aboriginal people - for sensitive and contact reasons - is another shortfall of the study design. CATI surveys are not suitable to conduct interviews with ATSI people as many ATSI do not have telephone access and live in remote communities. Most general household surveys that do include ATSI people in the surveys are those who live in the cities and large rural towns, and hence the results can be a gross under-representation of the ATSI population.

## Conclusion

The main strengths of this study are the representative population sample, the large sample size and the wide range of risk factors assessed. Further strengths are the decision not to limit interviewing to the English language and the wide age range of respondents, including children as young as ten years. Its main weakness is the low ascertainment proportion and the related potential for selection bias. Conventional household-based sampling via land-line telephones is becoming less effective due to technical and social changes. The use of other forms of data collection such as computer-assisted self-interviewing (CASI) and the internet have shown promise in collecting sensitive information [43,45,46] although determination of epidemiological based sampling frames is still of concern for web-based surveys. An effective probability based sampling design is critically important in epidemiological studies and is dependent upon knowing the probability of selection of all participants [40].

Collecting valid and reliable data is a paramount concern of health researchers and survey research methodologists. The challenge is to design cost-effective studies especially those associated with low-prevalence issues, and to balance time and convenience against validity, reliability, sampling, coverage, non-response and measurement error issues. This study is used to highlight the methodological issues and avenues taken to overcome the problems of interviewing adults and children on an important, low-prevalent and sensitive issue and has shown the value of CATI in collecting data on this type of topic. This study has provided important public health information on the population prevalence of self-injury in Australia and the relationship between NSSI and related risk factors and outcome measures. It will allow a range of hypotheses to be generated and tested, and preventive activities to be implemented.

## Abbreviations

ANESSI: Australian National Epidemiological Study of Self-injury; CASI: Computer Assisted Self Interviewing; CATI: Computer Assisted Telephone Interviewing; EWP: Electronic White Pages; NHMRC: National Health & Medical Research Council; NSSI: Non-suicidal Self-injury; SPSS: Statistical Package for Social Sciences

## Competing interests

The authors declare that they have no competing interests.

## Authors' contributions

AWT participated in the design and co-ordination of the study, drafted the manuscript. GM conceived the design of the study, gained the funding, participated in the coordination of the study, involved in the drafting of the manuscript. EDG participated in the design and co-ordination of the study, performed statistical analyses and involved in the drafting of the manuscript. SS participated in the design and co-ordination of the study, performed statistical analyses and involved in the drafting of the manuscript. SF participated in the co-ordination of the study, performed statistical analyses and involved in the drafting of the manuscript. PH participated in the design and co-ordination of the study, involved in the drafting of the manuscript. JH participated in the design and co-ordination of the study, involved in the drafting of the manuscript. All authors read and approved the final manuscript.

## Pre-publication history

The pre-publication history for this paper can be accessed here:

http://www.biomedcentral.com/1471-2288/11/20/prepub
